# P34 A case of haemophagocytic lymphohistiocytosis triggered by primary Epstein–Barr virus infection

**DOI:** 10.1093/rap/rkae117.065

**Published:** 2024-11-05

**Authors:** Shama Mahal, Erica Gurung, James Carroll, Rupak Moitra

**Affiliations:** Basingstoke and North Hampshire Hospital, Basingstoke, United Kingdom; Basingstoke and North Hampshire Hospital, Basingstoke, United Kingdom; Basingstoke and North Hampshire Hospital, Basingstoke, United Kingdom; Basingstoke and North Hampshire Hospital, Basingstoke, United Kingdom

## Abstract

**Introduction:**

Haemophagocytic lymphohistiocytosis (HLH) is an uncommon systemic hyperinflammatory state characterised by uncontrolled activation of the immune system. HLH may occur in a primary (familial) form, but more commonly occurs secondary to another disease process. Such triggers include infections, autoimmune disease and malignancy. We present the case of a 24-year-old female who was previously fit and well, who was diagnosed with HLH secondary to primary Epstein-Barr virus (EBV) infection.

**Case description:**

The patient was admitted with a 10-day history of fever, sore throat and abdominal pain. On examination she had bibasal crackles and epigastric and right upper quadrant tenderness. There was pharyngeal erythema but no exudate. No rash or joint inflammation.

Admission blood tests showed WBC 11.7 x10^9^/L, neutrophils 6.5 x10^9^/L, lymphocytes 4.3 x10^9^/L, platelets 142 x10^9^/L, CRP 179 mg/L, ALT 330 U/L, ALP 1022 U/L, bilirubin 37 mmol/L and lactate 1.81 mmol/L. A CT chest/abdomen/pelvis showed bibasal consolidation with small bilateral pleural effusions and mild splenomegaly.

The patient was hypotensive and tachycardic, and was spiking temperatures greater than 39 °C. She subsequently developed type 1 respiratory failure and was transferred to the intensive care unit. She was treated with broad-spectrum antibiotics for suspected sepsis secondary to pneumonia.

She remained unwell with high spiking fevers. HLH was suspected and ferritin was >100,000 mg/L with triglycerides 6.19 mmol/L, fibrinogen 1.1 g/L and LDH >1800 U/L. Her HScore was 200, indicating a high probability of HLH. Autoimmune screen including rheumatoid factor, ANCA and ANA were negative. A comprehensive infection screen identified a strongly positive EBV viral capsid antigen (VCA) IgM, positive EBV VCA IgG and negative EBV nuclear antigen (EBNA) with a viral load of 49,600 copies/ml. These results were consistent with acute primary EBV infection. Bone marrow biopsy confirmed haemophagocytosis.

A diagnosis of HLH triggered by primary EBV infection was made. The patient was discussed with the specialist HLH service at University College Hospital and was commenced on intravenous methylprednisolone and anakinra 200 mg twice daily. She was additionally given antivirals, antibiotics and antifungals as prophylaxis against secondary infection.

The patient improved clinically and biochemically. The dose of anakinra was reduced and eventually discontinued, and the patient was discharged on a tapering course of prednisolone.

**Discussion:**

HLH carries a high mortality rate and early recognition and treatment is vital. However, diagnosis is often delayed as HLH may resemble other conditions such as sepsis. Signs and symptoms of HLH include fever, hepatosplenomegaly, cytopenias, hyperferritinaemia, hypertriglyceridaemia, liver dysfunction and coagulopathy. There may be multi-organ dysfunction. The characteristic histopathological finding is haemophagocytosis on biopsy of bone marrow, lymph node, liver or spleen, however this may initially be absent.

EBV is the most common infective trigger for HLH, but it may occur with other viral and bacterial infections including tuberculosis. The most common autoimmune conditions associated with HLH include adult-onset Still’s disease (AOSD), systemic juvenile idiopathic arthritis and lupus. Haematological malignancy, in particular lymphoma, is another important cause and can sometimes be challenging to identify.

The initial presentation of AOSD can share many of the same clinical features as EBV infection, including fever, rash, sore throat, hepatosplenomegaly, lymphadenopathy and liver dysfunction. The rash of AOSD is classically salmon-pink coloured and comes and goes with the fever, and the fever typically occurs at the same time each day. In our patient, the absence of arthralgia, rash and leucocytosis meant AOSD was felt to be unlikely, but this was kept under review.

**Key learning points:**

• Early recognition and treatment of HLH is crucial to reducing mortality

• The HScore tool can be used to assess the probability of HLH

• Management of HLH involves supportive care, immunosuppression and treatment of the underlying cause

• Agents that may be used include steroids, cyclosporine, etoposide, anakinra and intravenous immunoglobulin

